# Imaging Retroviral RNA Genome Heterodimers Using Bimolecular Fluorescence Complementation (BiFC)

**DOI:** 10.3390/v17081112

**Published:** 2025-08-13

**Authors:** Eunice C. Chen, Rebecca K. Maldonado, Leslie J. Parent

**Affiliations:** 1 Department of Medicine, Division of Pulmonary, Allergy, and Critical Care Medicine, Penn State College of Medicine, Hershey, PA 17033, USA; exc281@psu.edu; 2 Department of Molecular & Precision Medicine, Penn State College of Medicine, Hershey, PA 17033, USA; rjk297@psu.edu; 3 Department of Medicine, Division of Infectious Diseases and Epidemiology, Penn State College of Medicine, Hershey, PA 17033, USA

**Keywords:** RNA genome dimer, retroviruses, RSV, RNA-RNA intermolecular interactions, bimolecular fluorescence complementation, confocal microscopy, fluorescence imaging

## Abstract

Retroviruses are single-stranded RNA viruses that package two copies of their positively stranded RNA genomes as a non-covalent dimer into newly formed virions. This process is evolutionarily conserved, and disruption of genome dimerization results in production of non-infectious virus particles. Genome dimers can be packaged as homodimers, containing two identical RNAs, or heterodimers, consisting of two genetically distinct copies. Genome dimerization generates genetic diversity, and different retroviruses have preferences for the type of genome dimers packaged into virions. We developed a novel imaging approach to specifically label and detect retroviral genome heterodimers in cells using a modified bimolecular fluorescence complementation (BiFC) technique. This method utilizes viral genomes encoding two different RNA stem-loop cassettes that each specifically binds to an RNA-binding protein conjugated to a split fluorophore. When two genetically different genomes are within close proximity, the fluorophore halves come together to reconstitute fluorescence. These BiFC-labeled RNA dimers can be visualized and tracked in living cells and interact with retroviral Gag proteins. This method has the advantage of low background fluorescence and can be applied to the study of dimeric or double-stranded RNAs of viruses and other organisms.

## 1. Introduction

Retroviruses are significant pathogens of humans and animals, causing a variety of cancers and immunodeficiency syndromes. Their single-stranded, positive-sense RNA genomes are packaged as non-covalently linked dimers into virions that are released from the plasma membrane of infected cells. Genome dimerization is conserved amongst all *Orthoretrovirinae*, including Rous sarcoma virus (RSV), human immunodeficiency virus (HIV), and murine leukemia virus (MLV). Genome dimerization plays several significant roles in the virus replication cycle, including regulating the translation of viral proteins, facilitating strand transfer reactions during reverse transcription, and generating diversity through recombination of genetically distinct RNAs, all of which are advantageous for the virus [1,2,3,4,5,6,7,8,9,10,11,12,13,14]. Disruption of genome dimerization or mutation of the dimer initiation site near the 5′ end of the genome results in production of noninfectious virus particles.

The characteristics of retrovirus genome dimerization have been explored in vivo over the years, specifically to examine whether retroviruses package genome heterodimers (two genetically distinct copies of the genome dimerize and are co-packaged) or genome homodimers (two genomes identical in sequence dimerize and undergo packaging) [13,15,16,17,18,19,20,21,22]. Moloney MLV genomes dimerize co-transcriptionally in the nucleus, resulting in the preferential packaging of genome homodimers [16,17,18,19]. In lentiviruses, such as HIV type 1, genome dimerization occurs in a random fashion and there is no preference for packaging genome heterodimers or homodimers [9,13,20,22]. In the *Alpharetrovirus* RSV, genome heterodimers have been visualized in the nucleus, cytoplasm, and at the plasma membrane, indicating that dimers may arise during or shortly after transcription [15]. Both heterodimers and homodimers were detected in RSV particles, although homodimers were preferentially packaged.

The above in vivo studies utilized single-molecule RNA labeling methods to visualize retroviral RNA genomes within cells and single virions. These methods include the use of RNA aptamer and stem-loop systems as well as single-molecule RNA fluorescence in situ hybridization (smFISH). In the RNA aptamer and stem-loop system, a cassette of RNA stem-loops is inserted into the RNA of interest, and these loops are bound with high affinity by a protein that has been fused to a fluorophore (reviewed in [23,24,25,26,27]). The original stem-loop system used is the MS2 coat protein (CP) system, derived from the MS2 bacteriophage [28,29,30,31,32,33], in which 24 copies of the MS2 stem-loop inserted into the RNA are bound by the fluorophore-labeled MS2 CP (MCP). The MS2-MCP complex can be visualized by microscopy throughout all cellular compartments, including the nucleus, as long as the MCP contains a nuclear localization signal [28]. RNA stem-loops such as these do not naturally occur in mammalian cells, so the fluorophore-tagged MCP will not label other mammalian RNAs [25]. However, there can be diffuse background fluorescence signal present when MCPs not bound to their cognate RNAs accumulate in the nucleus. Other stem-loop aptamer systems that have been developed include the BglG binding protein (Bgl), based on the *Escherichia coli* BglG protein [20], and the PP7 CP (PCP), derived from the PP7 bacteriophage [31,34]. Similarly to the MS2-MCP system, the fluorophore-tagged Bgl and PP7 proteins contain a nuclear localization signal to allow visualization of RNA-protein complexes within the nucleus and dispersed throughout the cell.

In this study, we used two different stem-loop aptamer systems combined with bimolecular fluorescence complementation (BiFC) to specifically visualize retroviral genome heterodimers within cells. We constructed plasmids to express the MCP fused to the N-terminal half of the Venus fluorophore (MS2-VN) paired with constructs expressing the C-terminal half of the Venus fluorophore fused to either Bgl binding protein (Bgl-VC) or PCP (PP7-VC). When two retroviral genomes containing distinct RNA aptamers bound to VN or VC are in close proximity, the Venus fluorophore will be reconstituted to generate fluorescence visible by microscopy [35,36,37,38,39]. Normally, fluorescently tagged MS2, Bgl, and PP7 proteins have diffuse signals in the cell unless bound to their respective RNA aptamers. With BiFC, there is less diffuse background signal because there will only be fluorescence when the proteins bind their target RNAs and the RNAs dimerize, reforming the Venus fluorophore. Using this approach, we demonstrated that BiFC-labeled genome dimers are visible as functional fluorescently labeled punctate foci that traffic through the cell and interact with viral Gag proteins. The BIFC labeling system for RNA labeling can be used in both fixed-cell imaging and live-cell imaging with minimal background fluorescence.

## 2. Materials and Methods

### 2.1. Plasmid Construction

The replication-competent RSV provirus construct (RC.V8) [40] was used as the backbone to generate a construct containing a cassette of either 24 MS2 loops (RC. MS2) [28], 18 BglG loops (RC. Bgl) [20], or 24 PP7 loops (RC. PP7) [31] inserted between *gag* and *pol* to specifically label unspliced viral RNA. pCR4-24XMS2SL-stable was provided by Dr. Robert Singer (Albert Einstein Medical College, Bronx, NY, USA) (Addgene plasmid # 31865; http://n2t.net/addgene:31865; RRID:Addgene_31865) [28], as was pCR4-24XPP7SL (Addgene plasmid # 31864; http://n2t.net/addgene:31864; RRID:Addgene_31864) [31]. The BglG loops were removed from GagCeFP-BglSL, a gift from Dr. Wei-Shau Hu (National Cancer Institute at Frederick, Frederick, MD, USA) [20]. These constructs were then modified to insert the *cfp* gene after the *gag* gene (RC.GagCFP.MS2, RC.GagCFP.Bgl, and RC.GagCFP.PP7) to encode an internally expressed GagCFP as previously described [15,41]. The MS2-YFP construct was a gift from Dr. Robert Singer, the Bgl-mCherry was a gift from Dr. Wei-Shau Hu. phage-ubc-nls-ha-tdPCP-gfp was provided by Dr. Robert Singer (Addgene plasmid # 40650; http://n2t.net/addgene:40650; RRID:Addgene_40650). The PP7 coat protein was moved into the Bgl-mCherry backbone and the fluorophore was swapped out. The Venus fluorophore was split according to a previously published strategy [42]. The N-terminal 173 amino acids of Venus were fused to either BglG binding protein (Bgl-VN) or PP7 CP (PP7-VN) and the C-terminal 155 amino acids of Venus were fused to MCP (MS2-VC). pDEST-Sun1-mCherry was a gift from Dr. Jan Karlseder (Salk Institute for Biological Studies, La Jolla, CA, USA) [43].

### 2.2. Cell Culture and Transfection

Experiments were performed in QT6 cells [44], a chemically transformed quail fibroblast cell line maintained in F10 medium supplemented with 10% fetal calf serum, 9% tryptose phostphate, 1% chicken serum, penicillin and streptomycin, and amphotericin B and cultured at 38.5 °C with 5% CO_2_. Cells were transfected with the calcium phosphate method [45]. For fixed-cell microscopy, cells were grown on glass coverslips. Twenty-four hours post-transfection, coverslips were fixed in 3.7% formaldehyde/PBS at room temperature, washed in PBS, 4′,6-diamidino-2-phenylindole (DAPI, MilliporeSigma, Rockville, MD, USA) stained, and mounted onto glass slides with Prolong diamond antifade (ThermoFisher Scientific, Waltham, MA, USA). For live-cell microscopy, cells were grown on glass-bottomed cell culture dishes (Mattek) prior to transfection, then washed once with 1x PBS, and phenol red-free imaging media was added prior to imaging (ThermoFisher Scientific, Waltham, MA, USA). For instances in which nuclei were labeled with DRAQ5 (ThermoFisher Scientific, Waltham, MA, USA), cells were incubated with primary growth medium containing DRAQ5 for 1 h at 37 °C.

### 2.3. Microscopy, Image Acquisition, and Image Analysis

Microscope images were obtained on a SP8 confocal microscope (Leica Microsystems, Wetzlar, Germany) using a 63x oil-immersion objective, and UV, white light, and argon lasers. Z-stacks were obtained with thickness of 0.3 μm through the width of the nucleus (as indicated by DAPI staining). Live-cell imaging was performed with the same microscope using a heated live-cell stage with a 63x water-immersion objective.

Images presented in Figures 2B,C and 3A were subjected to Huygens Professional 24.10 deconvolution (Scientific Volume Imaging, Hilversum, Netherlands) using the classical maximum likelihood estimation (CMLE) algorithm. Further image analysis and processing was completed using Imaris 10.2.0 (Bitplane an Oxford Instruments Company, Belfast, United Kingdom). Z-stacks were used to create cross-sections of the x, y; z, y; and x, z dimensions of the cell. Co-localization was assessed via signal-based analysis using Imaris and displayed as a white co-localization channel. The Imaris spot function was used to identify Gag, RNA dimers, and/or co-localized foci and to perform particle tracking. Histograms of all images were adjusted and images that were not deconvolved were subjected to a Gaussian filter.

## 3. Results

### 3.1. Visualization of Genome Heterodimers Within the Cell

**Figure 1:**
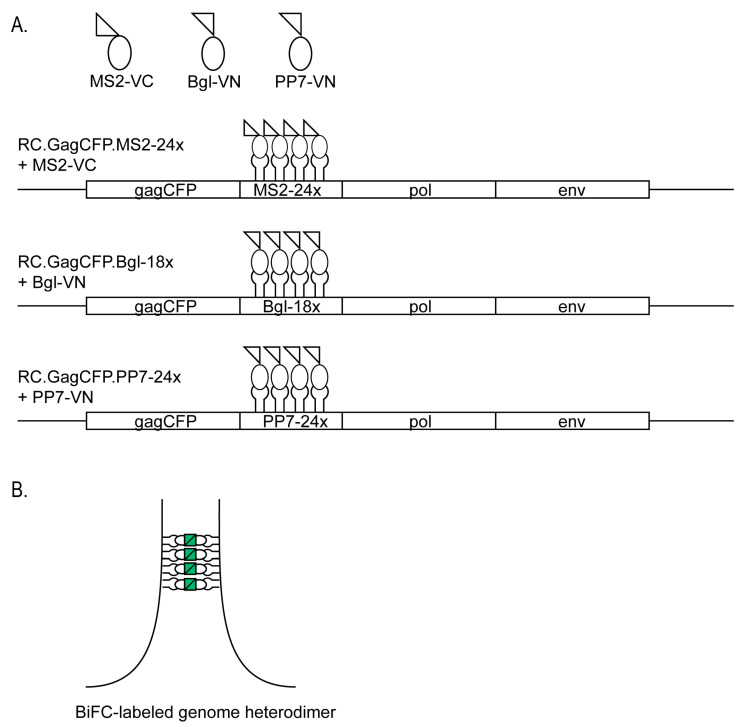
Overview of the bimolecular fluorescence complementation (BiFC) method used to visualize RSV genome dimers. (**A**) Schematic of the constructs used in this study. The MS2 CP was fused to the C-terminus of Venus to create MS2-VC, while Bgl binding protein or PP7 CP were fused to the Venus N-terminus (Bgl-VN and PP7-VN). Three RC.V8 constructs were generated to express GagCFP in *cis* and contained either 24 MS2 stem-loops (RC.GagCFP.MS2-24x), 18 Bgl stem-loops (RC.GagCFP.Bgl-18x), or 24 PP7 stem-loops (RC.GagCFP.PP7-24x). The split-fluorophore tagged proteins specifically bind their respective stem-loops. (**B**) When RC.GagCFP.MS2-24x bound to MS2-VC dimerized with either RC.GagCFP.Bgl-18x bound to Bgl-VN or RC.GagCFP.PP7-24x bound to PP7-VN, the Venus fluorophore will be reconstituted allowing for fluorescence to occur. In the absence of RNA binding, the coat proteins do not fluoresce (see Supplementary Figure S1).

**Figure 2:**
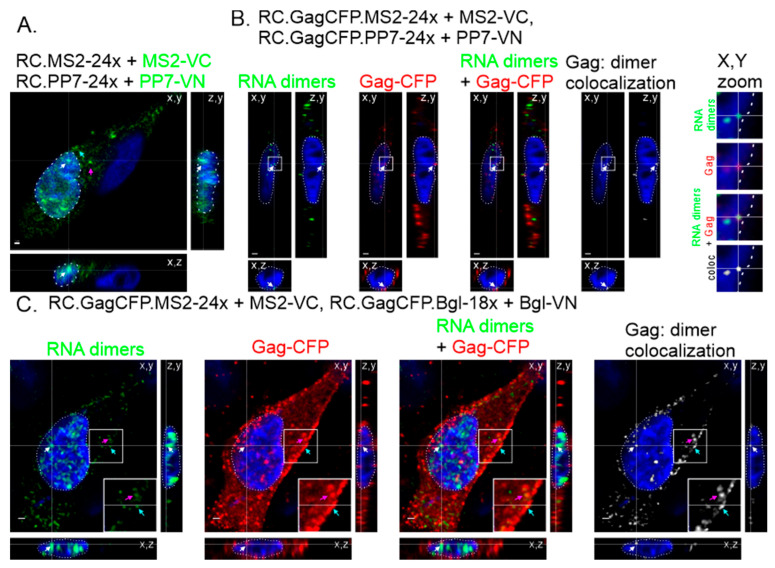
Labeling RSV genome heterodimers in fixed cells. (**A**) QT6 cells were transfected with RC.MS2-24x, MS2-VC, RC.PP7-24x, and PP7-VN. The RC.MS2-24x and RC.PP7-24x constructs did not express an internal Gag-CFP fusion protein. Z-stacks were collected via confocal microscopy and used to generate a cross-section. RNA genome heterodimers appeared as green foci throughout the cell. The white crosshairs and arrow indicate an RNA genome heterodimer in the nucleus (DAPI, dashed white outline). Foci were also observed in the cytoplasm (magenta arrow) and at the plasma membrane (cyan arrow). (**B**) Z-stacks of cells expressing RC.GagCFP.MS2-24x + MS2-VC and RC.GagCFP.PP7-24x + PP7-VN were used to generate cross-sections. An RNA genome heterodimer (green, left panel) was seen in the nucleus (DAPI, dashed white outline) as indicated by the white cross-hairs and arrow. The RC.V8 genome constructs express Gag fused to CFP (red). A Gag-genome heterodimer complex was observed in the nucleus (center panel, white arrow). A colocalization channel was generated and revealed Gag-genome heterodimer complexes (white) in the nucleus, cytoplasm and the plasma membrane (right panel). The focus of interest is outlined in the white square and included in the X,Y zoom in (right). (**C**) Cells co-expressing RC.GagCFP.MS2-24x + MS2-VC and RC.GagCFP.Bgl-18x + Bgl-VN (left panel) contained RNA genome heterodimers (green) in the nucleus (white arrow), cytoplasm (magenta arrow), and plasma membrane (green arrow). When Gag-CFP was visualized (red), Gag-genome heterodimer complexes were visualized in the nucleus, cytoplasm, and plasma membrane (white, magenta, and green arrows, respectively; center panel). Cytoplasmic and plasma membrane complexes are included in the inset. A colocalization channel was generated (white, right panel) revealing Gag-genome heterodimer complexes in the nucleus (white arrow), cytoplasm (magenta arrow), and at the plasma membrane (cyan arrow). Nuclear localization was confirmed by three-dimensional cross-sections of Z-stacks in the x,y; x,z; and z,y planes. Scale bar = 1 µm.

To detect intracellular genomic RNA heterodimers, we co-transfected two genetically distinct retroviral genomes containing MS2 and Bgl RNA stem-loops or MS2 and PP7 RNA stem-loops with their respective binding proteins fused to a split Venus fluorophore (VN or VC). A schematic diagram of the viral constructs is shown in Figure 1A. The RNA stem-loops are located between *gag* and *pol* and therefore will only label unspliced viral RNAs, which are packaged into virions as the viral genome. Fluorescence is visible when the genomes are in close proximity, allowing the Venus fluorophore to reform (Figure 1B). To check for possible nonspecific background fluorescence, we co-expressed either PP7-VN or Bgl-VN with MS2-VC without their respective RNA stem-loops, and BiFC signal was not produced (Supplemental Figure S1). RSV genome constructs containing MS2 loops and PP7 loops with their respective split fluorophore-fused binding proteins were co-transfected into quail fibroblast QT6 cells, fixed with paraformaldehyde, and then imaged by confocal microscopy. Fluorescent Venus foci representing BIFC-labeled genome heterodimers were visible in the nucleus, cytoplasm, and along the plasma membrane (Figure 2A). A cross-section was generated from a Z-stack with cross-hairs (white arrows) marking a genome heterodimer, which can be seen in the nucleus (DAPI, white outline) in three dimensions. Genome heterodimers were also observed in the cytoplasm (magenta arrow) and appeared to be located along the plasma membrane (cyan arrow). Each fluorescent focus represented one or more genome heterodimers, composed of two genetically distinct retroviral RNA genomes, consistent with our previous studies showing that RSV genome heterodimers were present in all three locations in the cell [15]. When the BIFC-labeled MS2 (RC.GagCFP.MS2-24x + MS2-VC) and PP7 (RC.GagCFP.PP7-24x + PP7-VN) genome heterodimers (green) were visualized with Gag-CFP (red), colocalization (white) of genome heterodimers with Gag-CFP was seen within the nucleus (DAPI, white outline), as shown in three dimensions (Figure 2B). Similar results were obtained when MS2 (RC.GagCFP.MS2-24x + MS2-VC) and Bgl (RC.GagCFP.Bgl-18x + Bgl-VN) heterodimers (green) were expressed with Gag-CFP (red), and co-localized foci (white) were observed in the nucleus, cytoplasm, and at the plasma membrane (Figure 2C). Together these results demonstrated that both BiFC RNA stem-loop systems (MS2 + PP7 and MS2 + Bgl) were effective at labeling genome heterodimers in fixed cells.

### 3.2. Live-Cell Imaging of BiFC-Labeled Genome Heterodimers in the Nucleus

**Figure 3:**
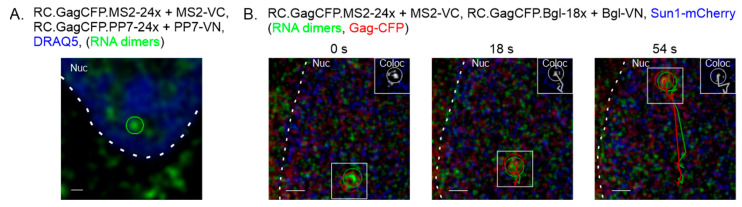
Live-cell imaging of nuclear RSV genome heterodimers. (**A**) QT6 cells were transfected with plasmids expressing RC.GagCFP.MS2-24x, RC.GagCFP.PP7-24x, PP7-VN, and MS2-VC. DRAQ5 was used to visualize the nucleus (blue). A genome heterodimer was tracked in the nucleus within a single confocal z-slice imaged approximately every 3 s. In a still image obtained at 24 s, a genome heterodimer (green focus) was visible within the nucleus (blue, dashed line). (**B**) QT6 cells were transfected with plasmids encoding RC.GagCFP.MS2-24x, RC.GagCFP.Bgl-18x, Bgl-VN, and MS2-VC. Sun1-mCherry was used to mark the inner leaflet of the nuclear membrane (blue). A complex containing RNA genome dimer (green) with Gag-CFP (red) colocalized in the nucleus and trafficked together. A colocalization channel (white) is shown in the upper right corner of the image, indicating that the red and green foci were overlapping. Scale bar = 0.5 µm.

Next, the BiFC system was used in living cells to visualize and characterize RSV genome heterodimers. QT6 cells were co-transfected with plasmids expressing RC.GagCFP.MS2-24x, RC.GagCFP.PP7-24x, MS2-VC, and PP7-VN. Cells were stained with DRAQ5 (blue) to visualize the nucleus (nuclear DNA) and live-cell microscopy was performed at a rate of approximately 3 frames sec^−1^. An individual frame extracted from the imaging series shows a genome heterodimer in the nucleus (Figure 3A). In a subsequent experiment, Sun1-mCherry was co-transfected to mark the inner leaflet of the nuclear membrane (white dashed line) and QT6 cells were co-transfected with plasmids expressing RC.GagCFP.MS2-24x, RC.GagCFP.Bgl-18x, MS2-VC and Bgl-VN (Figure 3B). A genome heterodimer (green) and Gag (red) were visualized moving together inside the nucleus. Particle tracking was performed using Imaris software (indicated by red and green overlapping circles) at times 0, 18 and 54 secs. The visualization of a genome heterodimer: Gag complex within the nucleus suggests that genome dimerization in RSV can occur in the nucleus, as previously demonstrated [15], and dimers can be recognized by the nuclear Gag protein to form a viral ribonucleoprotein complex.

### 3.3. BiFC-Labeled Genome Heterodimers Co-Localize with GagCFP in the Cytoplasm and at the Plasma Membrane

**Figure 4:**
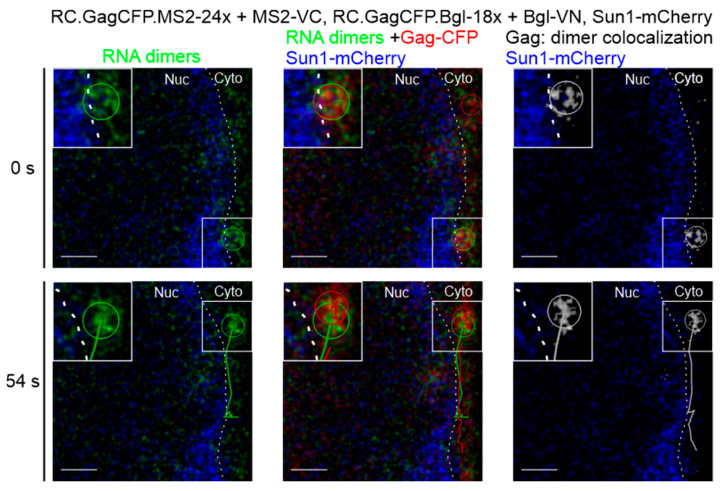
Live-cell imaging of RSV Gag-genome heterodimer complexes. QT6 cells were transfected with RC.GagCFPMS2-24x, MS2-VC, RC.GagCFP.Bgl-18x, and Bgl-VN. Sun1-mCherry was used to outline the inner nuclear membrane (blue). A single confocal Z-slice was imaged approximately every 3 s. RNA heterodimers appeared as green signal and form discrete foci. An RNA heterodimer (green line) and a Gag-CFP focus (red line) trafficked together in the cytoplasm (cyto) near the nuclear rim over time. A colocalization channel (white) was generated and tracked (white circle and line). Zoomed in images of the particles are included to show more detail (inset). Scale bar = 1 µm.

BiFC-labeled genome heterodimers were visualized co-localizing with Gag-CFP in the fixed cells shown in Figure 2. We sought to further characterize dynamic interactions of genome heterodimers and Gag using live-cell microscopy (Figure 4, Supplementary Videos S1–S3). QT6 cells were co-transfected with plasmids expressing retroviral genomes containing MS2 or Bgl RNA stem-loops and a Gag-CFP fusion protein (RC.GagCFP.MS2-24x and RC.GagCFP.Bgl-18x), the split fluorophore-tagged binding proteins (MS2-VC and Bgl-VN), and Sun1-mCherry to label the inner nuclear membrane. A single Z-plane was imaged approximately every 3 s. Colocalization between BiFC-labeled genome heterodimer foci (green) and Gag-CFP (red) was assessed via the generation of a colocalization channel (white). We then performed particle tracking of the heterodimers (green), Gag-CFP (red), and the colocalized signal (white). We focused on colocalized genome heterodimers and Gag-CFP foci that remained together throughout the duration of the movie (54 s), as demonstrated by the green (dimer), red (Gag), and white (colocalization) tracks in Figure 4. The Gag-genome heterodimer complex trafficked together over time in the cytoplasm, along the outer aspect of the nuclear membrane (labeled by Sun1 in blue and indicated by a white dashed line). The insets show an enlarged image of the tracked co-localized foci containing Gag and genomic RNA foci.

## 4. Discussion

We present a novel BiFC technique to selectively visualize retroviral genome heterodimers within fixed and living cells. We designed three-separate RSV constructs containing RNA stem-loop cassettes derived from MS2, Bgl, or PP7 that bind their cognate RNA binding proteins (MCP, Bgl, or PCP). The RNA binding proteins were fused to either the N-terminal or C-terminal half of the Venus fluorophore (MS2-VC, Bgl-VN, or PP7-VN). In this BIFC system, fluorescence was only visible when the genomes were within close proximity, allowing the Venus fluorophore to refold (Figure 1). The fluorescent foci visualized represent RSV genomic RNA heterodimers. When the genomic RNA constructs and their respective fluorophore-labeled binding proteins were visualized in fixed cells, BiFC-labeled genome heterodimers were seen within the nucleus, in the cytoplasm, and at the plasma membrane (Figure 2), recapitulating the intracellular pathway for RSV particle assembly, similar to our previous study [15]. When the binding proteins were expressed alone, without their respective RNA aptamer-containing RSV genomes, no BiFC fluorescence was visualized (Supplemental Figure S1). Using live-cell microscopy, we visualized BiFC-labeled genome heterodimers in complex with Gag proteins moving within the nucleus and in the cytoplasm (Figure 3 and Figure 4, Supplementary videos S1–S3). Together, these findings demonstrate that BiFC-labeled genome heterodimers maintained the typical trafficking pathway and function of a genome dimer and were bound by the Gag protein. From these experiments, we cannot discern whether more than a single genome dimer was present in each of the Venus-labeled RNA puncta, and additional methods would be needed to attempt to estimate the number of RNA molecules present in each focus. This study provides evidence for the use of BiFC as a means to visualize retroviral genome heterodimers trafficking within a cell and in complex with the viral Gag protein. We believe this technique opens the door to further characterize genome heterodimer formation and dynamic movement during retroviral genome packaging and virion assembly. In addition, a similar BIFC approach could be adapted for the study of double-stranded viruses and cellular processes involving RNA multimers [46,47].

There are many benefits to using a BiFC-labeling system for the study of RNA dimers and multimers. One notable improvement over other detection systems is the decrease in background fluorescence from unbound fluorescently tagged binding proteins. In traditional RNA stem-loop and binding protein systems, the binding protein contains a nuclear localization signal and generates a low level of background fluorescence in the nucleus. However, in the BiFC-labeling system, fluorescence is only visible when the fluorophore halves are able to reform, resulting in less diffuse background fluorescence. Importantly, we showed that expression of the coat proteins alone without their respective RNA stem-loops did not result in visible fluorescence (Supplementary Figure S1). This result also suggests that the RNA binding proteins fused to Venus VN and VC do not produce pre-formed fluorescent signals prior to binding their cognate RNA stem-loops. Additionally, the MS2 and PP7 stem-loops have been shown to bind their binding proteins in a co-transcriptional fashion [28]. This idea is supported by the finding that fluorescently tagged BiFC genome heterodimer foci were visualized in nuclei, as demonstrated in an earlier study using a different detection system [15]. Therefore, it is more likely that the stem-loop containing genomes bind their respective binding proteins first and then dimerize when the RNAs move into close approximation, resulting in refolding of the Venus fluorophore.

Importantly, the BiFC-labeling system is flexible in terms of the RNA stem-loop cassettes utilized and compatibility with live-cell imaging. We could detect BiFC-labeled genome heterodimer formation using both the MS2/Bgl stem-loop and MS2/PP7 stem-loop systems, allowing us to visualize and characterize genome heterodimers as they functioned within living cells. These genome heterodimers were observed moving within different subcellular compartments (Figure 2, Figure 3 and Figure 4), suggesting the inclusion of RNA stem-loops and binding of the MCP, BglG, and PCP did not interfere with trafficking of the viral RNA genome. Additionally, we visualized genome heterodimers that colocalized and trafficked with Gag-CFP in living cells (Figure 3 and Figure 4, Supplementary Videos S1–S3), suggesting the inclusion of RNA stem-loops and binding of the VN and VC proteins did not preclude viral RNA:Gag protein interactions.

Of note, the PP7 binding protein does undergo nucleolar localization [48], although this quality does not appear to interfere with visualization of genome heterodimer in other parts of the cell. However, this point is important to keep in mind if the RNA of interest is predominantly localized to nucleoli, as there may be an unacceptable level of background fluorescence. Additionally, the BiFC interaction is irreversible once the fluorophore has reformed following interaction of the molecules of interest. This property can be helpful when studying transient interactions, as it allows for stabilization of brief binding events. However, because the interaction is locked in, there may be overrepresentation or skewing of downstream events from a transient interaction, especially when used in live-cell imaging. For example, in the case of dynamic interactions, once the binding between the two molecules occurs, this initial complex could be overrepresented and could prevent binding of new partners. It is important to keep these considerations in mind when employing BiFC-labeling to be certain that the characteristics of the system are applicable to the unique traits of the molecules of interest.

In recent years, there has been an increased interest in RNA-RNA interactions (RRI) in areas such as gene expression regulation, RNA metabolism, and RNA virus replication (reviewed in [49]). These interactions can be intermolecular (between two different RNA molecules) or intramolecular (between two distinct regions of the same RNA molecule). Numerous innovative biochemical and imaging techniques have been developed to allow researchers to detect and map RRIs (reviewed in [49]). Additionally, there have been many new methods to visualize single-RNA molecules using live-cell microscopy (reviewed in [27]). We present BiFC as an additional technique to further characterize known RRIs within fixed and living cells. As we have demonstrated, the strength of BiFC-labeling of RRIs is the ability to visualize subcellular localization and interaction with other host or viral proteins in both fixed and living cells with minimal background fluorescence.

Insertion of long RNA stem-loop cassettes can potentially affect structure, function, of transport of RNAs and should be considered when designing experiments to study retroviral genomes. Importantly, these aptamers have been used to study retroviral RNA trafficking and encapsidation in several previous publications, as reviewed recently [50]. However, we and others have shown that the addition of RNA stem-loops does not appear to interfere significantly with the trafficking and packaging of retroviral RNAs [13,15,20,41,51,52,53], and if placed in a nonessential region, infectivity can be maintained [41]. Importantly, the ability to visualize BiFC-tagged RNA dimers within living cells allows the further characterization of RNA-RNA intermolecular interactions in a novel way. Because “seeing is believing” [54] when it comes to RNA-RNA intermolecular interactions, this technique can be the starting point to developing new experiments to better understand their lifespan and function.

## Data Availability

Data and reagents will be made available upon request. Correspondence and requests for materials can be addressed to Leslie J. Parent.

## Supplementary Materials

The following supporting information can be downloaded at: https://www.mdpi.com/article/10.3390/v17081112/s1, Video S1: Trafficking of heterodimer in the cytoplasm; Video S2: Gag-RNA heterodimer co-trafficking in the cytoplasm, merged images of Gag and RNA channels from Video S1.; Video S3: Gag-heterodimer co-localization channel and particle tracking from Video S3; Video S4: A white colocalization channel was generated of RSV Gag-genome heterodimer complexes presented in Supplementary Videos 2 and 3; Figure S1: Expression of coat proteins without their respective RNAs.

## Abbreviations

The following abbreviations are used in this manuscript:

BiFC	Bimolecular fluorescence complementation
RSV	Rous sarcoma virus
HIV	Human immunodeficiency virus
MLV	Murine leukemia virus
smFISH	Single-molecule RNA fluorescence in situ hybridization
CP	Coat protein
MCP	MS2 coat protein
Bgl	BglG system
PCP	PP7 coat protein
RRI	RNA-RNA interactions
