# Supplementary material for: Imaging Retroviral RNA Genome Heterodimers Using Bimolecular Fluorescence Complementation (BiFC)
Source: Viruses. 2025 Aug 13;17(8):1112. doi: 10.3390/v17081112 (PMC12390704; doi:10.3390/v17081112)
Supplement: Supplementary file 1 [file viruses-17-01112-s001.zip › Supplemental Movies/SupplementaryVideoCaptionv2.pdf]

Supplementary Videos

Supplementary Video 1. Live-cell imaging of genome heterodimers in the nucleus. Quail QT6 cells were co-transfected with RC.MS2-24x, RC.PP7-24x, MS2VN-IRES-PP7VC, and Sun1-mCherry then imaged with live cell microscopy to visualize genome heterodimers. A single confocal slice was imaged approximately every 5 seconds. Shown in this video clip is a zoom in of three frames showing a genome heterodimer (green foci, white circles) traversing the nuclear rim (Sun1-mCherry, blue) into the cytoplasm. Scale bar = 0.5 µm

Supplementary Video 2. Live-cell imaging of RSV Gag-genome heterodimer complexes. Quail QT6 cells were co-transfected with RC.GagCFP.MS2-24x, RC.GagCFP.Bgl-18x, MS2-VC, Bgl-VN, and Sun1-mCherry then imaged with live-cell microscopy. A single confocal slice was imaged approximately every 3 seconds and this video is showing 54 seconds of a 17 minute movie. A heterodimer focus (green) was tracked (green circle) over time in the cytoplasm next to the nuclear membrane (Sun1-mCherry, blue). Scale bar = 1 µm

Supplementary Video 3. When the Gag-CFP channel (red) was overlaid with the RNA channel (green) of the cell displayed in Supplementary Video 2. We then performed particle tracking of Gag (red) and genome heterodimer (green) to visualize their movement throughout the cytoplasm together. The foci traveled along the nuclear membrane into the cytoplasm, presumably in the process of genome packaging. The complex appeared to merge with another Gag focus in the cytoplasm. Scale bar = 1 µm

Supplementary Video 4. A white colocalization channel was generated of RSV Gag-genome heterodimer complexes presented in Supplementary Videos 2 and 3. Scale bar = 1 µm
