# Supplementary figures and images for: Imaging Retroviral RNA Genome Heterodimers Using Bimolecular Fluorescence Complementation (BiFC)
Source: Viruses. 2025 Aug 13;17(8):1112. doi: 10.3390/v17081112 (PMC12390704; doi:10.3390/v17081112)

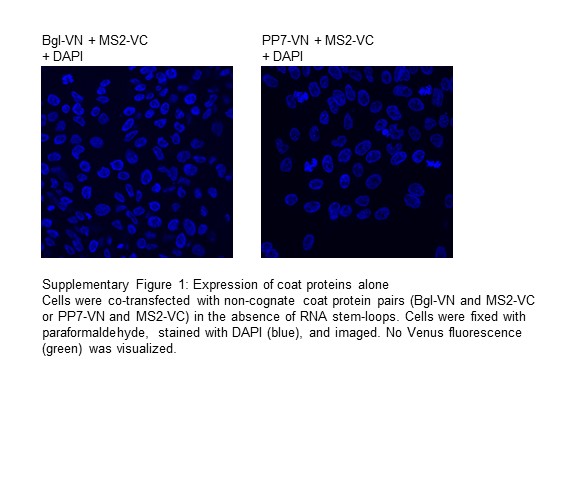

Supplement: Supplementary file 1 [file viruses-17-01112-s001.zip › Supplemental Figure/Supplemental Figure S1.jpg]
